# Adeno-associated virus 2/9 delivery of Cre recombinase in mouse primary afferents

**DOI:** 10.1038/s41598-018-25626-y

**Published:** 2018-05-09

**Authors:** Khaled Abdallah, Francis Nadeau, Francis Bergeron, Sylvie Blouin, Véronique Blais, Kelly M. Bradbury, Christine L. Lavoie, Jean-Luc Parent, Louis Gendron

**Affiliations:** 1 Département de pharmacologie-physiologie, Université de Sherbrooke, Sherbrooke, Québec Canada; 2 Département de médecine, Université de Sherbrooke, Sherbrooke, Québec Canada; 3 Département d’anesthésiologie, Université de Sherbrooke, Sherbrooke, Québec Canada; 4 Institut de pharmacologie de Sherbrooke, Université de Sherbrooke, Sherbrooke, Québec Canada; 5 Faculté de médecine et des sciences de la santé, Université de Sherbrooke, Sherbrooke, J1H 5N4 Québec Canada; 6 Bishop’s University, Sherbrooke, Québec Canada; 7 Centre de recherche du CHUS, Sherbrooke, Québec Canada; 8 Quebec Pain Research Network, Sherbrooke, Québec Canada

## Abstract

Genetically-modified animal models have significantly increased our understanding of the complex central nervous system circuits. Among these models, inducible transgenic mice whose specific gene expression can be modulated through a Cre recombinase/LoxP system are useful to study the role of specific peptides and proteins in a given population of cells. In the present study, we describe an efficient approach to selectively deliver a Cre-GFP to dorsal root ganglia (DRG) neurons. First, mice of different ages were injected in both hindpaws with a recombinant adeno-associated virus (rAAV2/9-CBA-Cre-GFP). Using this route of injection in mice at 5 days of age, we report that approximately 20% of all DRG neurons express GFP, 6 to 8 weeks after the infection. The level of infection was reduced by 50% when the virus was administered at 2 weeks of age. Additionally, the virus-mediated delivery of the Cre-GFP was also investigated via the intrathecal route. When injected intrathecally, the rAAV2/9-CBA-Cre-GFP virus infected a much higher proportion of DRG neurons than the intraplantar injection, with up to 51.6% of infected lumbar DRG neurons. Noteworthy, both routes of injection predominantly transduced DRG neurons over spinal and brain neurons.

## Introduction

Understanding brain and spinal cord circuits and how they influence physiological functions pose a major challenge. Identifying and studying the role of given peptides and proteins in a specific cell type is even more problematic, as most of them are expressed in many brain regions where they have different functions. In this context, genetically modified mice have helped over the past 20 years to elucidate the roles of many of these proteins, with gene knockout still being a widely used approach to identify functions of numerous peptides and proteins *in vivo*. Furthermore, genetically modified mouse models have also helped map local and complex neuronal circuitries. In particular, the use of Cre recombinase driver mice together with Cre-dependent (Cre-Lox) reporter mice or Cre-dependent viruses have helped better define various brain circuits and their roles^1,2^. Recently, numerous Cre-dependent transgenic mice models aiming at controlling the expression of endogenous peptides and proteins have been generated. Using conditional knockout (cKO) and conditional knockin (cKI) animal models, one can now study specific roles of peptides and proteins under physiological and pathophysiological conditions by ablating or inducing their expression in specific cell types in distinct brain regions or other organs.

Cre-dependent transgenic mice models require the expression of a Cre recombinase. Over the years, delivery systems to transfer genes to the central nervous system (CNS) have been widely investigated (for a review see ref.^3^). In addition to other vehicles, recombinant adeno-associated viruses (rAAVs) have proven their efficacy at delivering genes to the CNS. This was illustrated by rAAV-assisted gene therapy that successfully reversed disease phenotypes and restored normal functions in many models of neurodegenerative disorders (for reviews see refs^4,5^). Interestingly, the ability of a rAAV to infect a given type of cells is highly dependent on the viral capsid serotype^6^. The level of rAAV infection, i.e. the percentage of infected cells, is also dependent on the route of administration, the targeted tissue, as well as the animal’s age and the time post-injection^7,8^. As an example, it was shown that an i.c.v.-injected rAAV can achieve a much broader distribution of infection in the CNS when administered a few hours after birth compare to postnatal day 2^7,9^.

With respect to the pain pathways, dorsal root ganglia (DRG) neurons are of a particular interest. These highly diverse and specialized neurons are responsible for the transmission of nociceptive and proprioceptive signals from the periphery to the CNS and therefore play an essential role in the sensory system^10^. The importance of being able to manipulate these neurons *in vivo* to study their roles in pain processing is unquestionable. However, it can be challenging to specifically target these neurons.

Infection of DRG neurons has been reported by many studies^11–17^. It appears that one of the most effective routes for primary afferent infection is a direct delivery with repeated injections in the DRGs^15,18^. This approach is not only time consuming, but very challenging and ultimately invasive. However, the intrathecal administration of rAAVs was also shown to infect a high proportion of DRG neurons^11^. This route of injection is safe, rapid, minimally invasive and easy to perform in conscious mice by an experimented individual.

The aim of the present study was to develop an approach to specifically deliver a Cre recombinase to a maximum number of DRG neurons in mice as a tool to abolish (cKO) or to induce (cKI) expression of a protein in a Floxed mouse model. The rationale of such an approach being the possibility of studying the role of specific proteins (e.g. opioid receptors) in these neurons. The level and specificity of infection of primary afferents following subcutaneous (in the hindpaws) and intrathecal injection of the rAAV2/9-CBA-Cre-GFP virus were investigated.

## Results

### Intraplantar AAV infections

**Figure 1:**
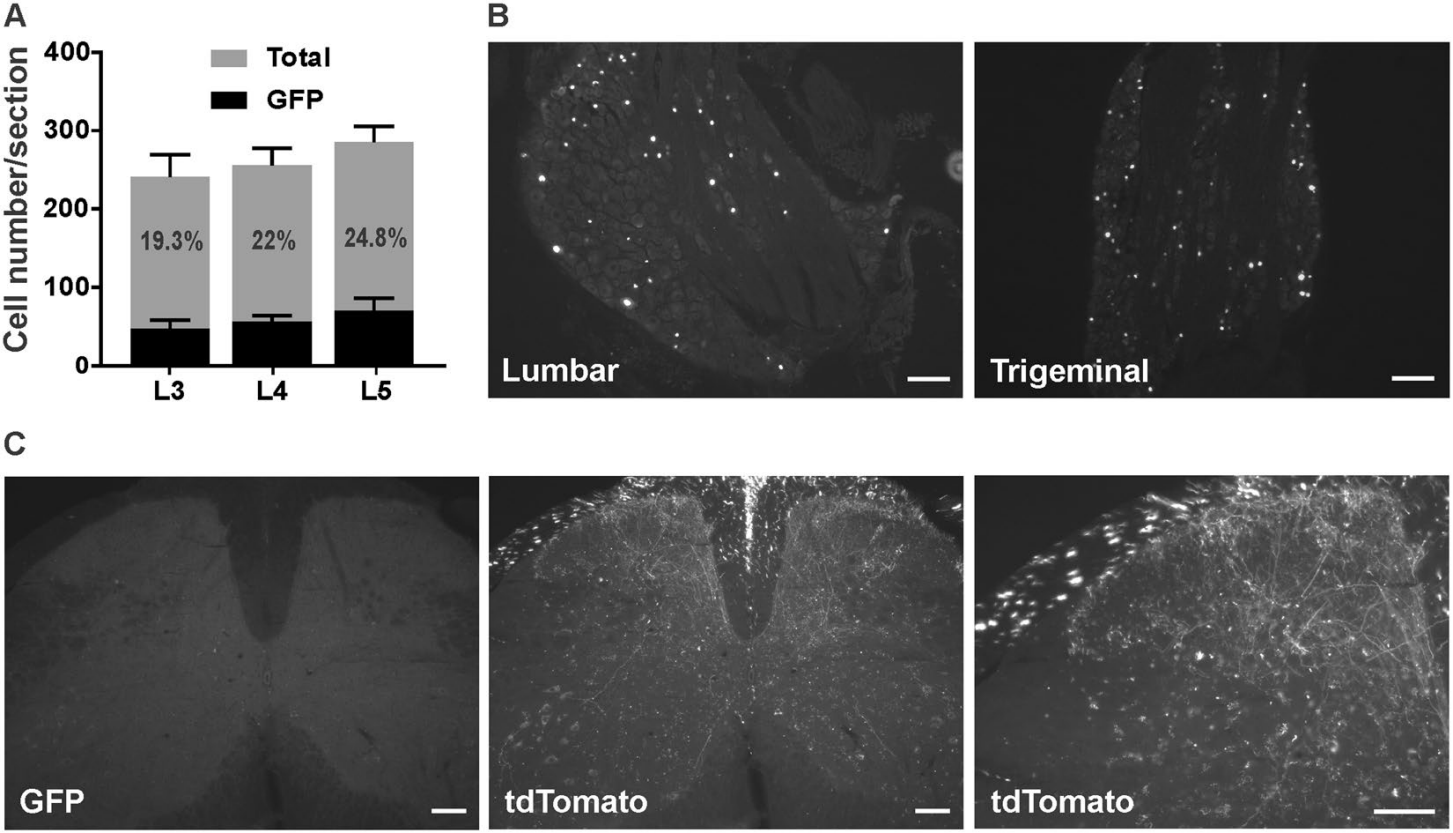
Distribution of GFP-positive cells in lumbar DRGs and spinal cord following intraplantar administration of AAV2/9-CBA-Cre-GFP at postnatal day 5. (**A**) Quantification of GFP positive cells in lumbar dorsal root ganglia (L3-5) following the intraplantar delivery of AAV2/9-CBA-Cre-GFP. Data are the mean +/− S.E.M. of 3 mice for which the % of GFP-labeled neurons observed in 8 to 18 sections per mice has been averaged. (**B**) Representative photomicrographs illustrating GFP distribution in lumbar and trigeminal ganglia. (**C**) Representative photomicrographs illustrating GFP (absence of) and tdTomato staining in the lumbar spinal cord of an animal co-injected with AAV2/9-CBA-Cre-GFP and emCBA-Flex-tdTomato-WPRE are shown. Scale bars = 100 µm.

**Figure 2:**
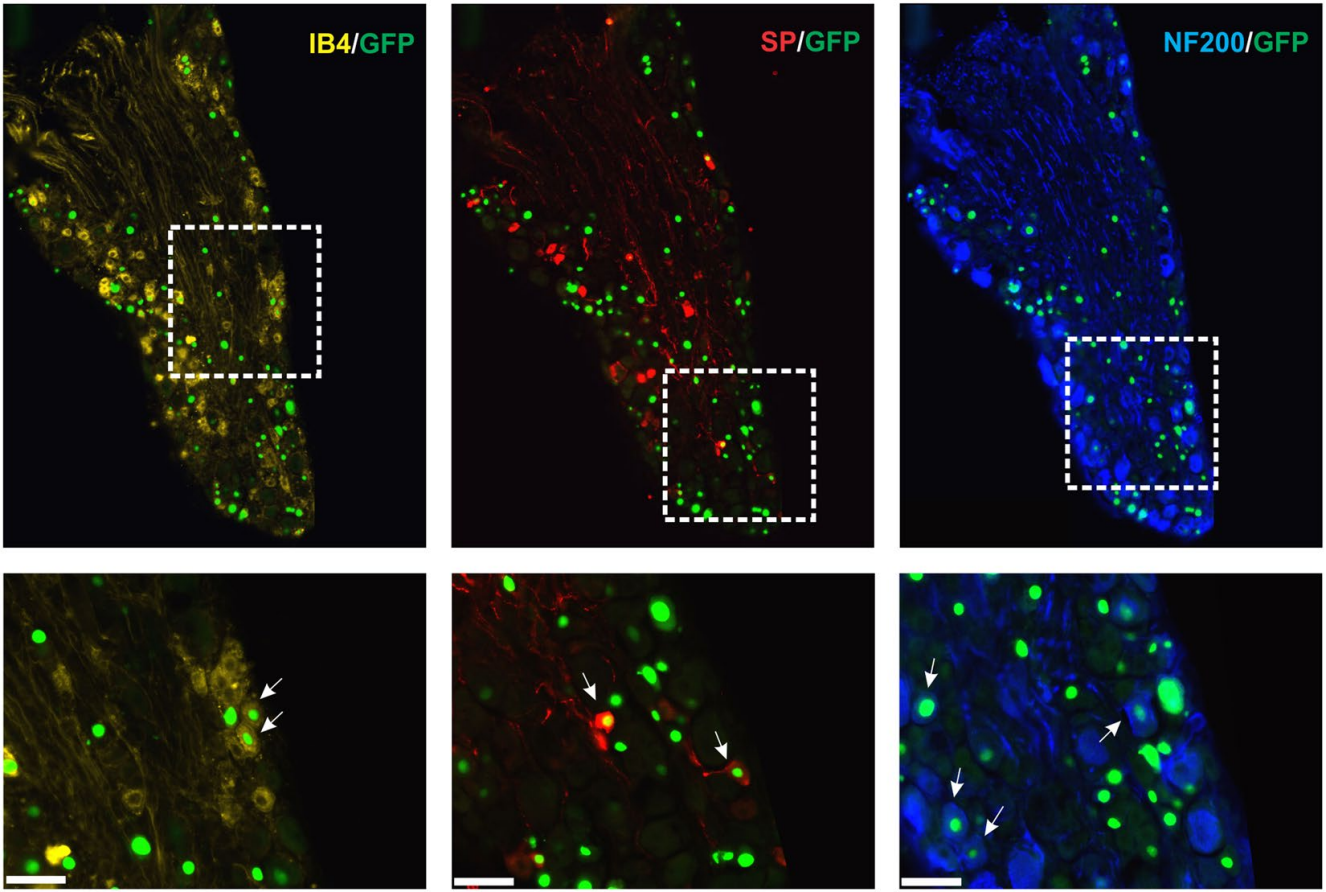
Distribution of the Cre-GFP in the neuronal subpopulations within the lumbar dorsal root ganglia. Representative photomicrographs showing co-localization of GFP with markers of peptidergic (Substance P), non-peptidergic (Isolectine B4) and large diameter myelinated (NF200) neurons are shown for mice (n = 6) injected in the plantar surface of both hindpaws with the AAV2/9-CBA-Cre-GFP virus. Arrows indicate neurons co-labeled for GFP and the indicated marker. Scale bars = 50 µm.

As illustrated in Fig. 1A,B, mice injected with the AAV2/9-CBA-Cre-GFP adenovirus at postnatal day 5 show a moderate level of transduction of lumbar dorsal root ganglia (DRG) neurons. Indeed, the nuclear GFP signal was observed in ~20% of total neurons (19.3 ± 3.8% in L3, 22 ± 1% in L4 and 24.8 ± 4.1% in L5; n = 3; Fig. 1A,B). Surprisingly, GFP-positive cells were also found in DRGs at other segmental levels (not shown) as well as in trigeminal ganglia (TGs) (Fig. 1B). A comparative analysis (Fig. 2, n = 6 mice) further revealed that the intraplantar adenovirus preferentially infected large myelinated (identified by the co-labelling of GFP and NF200, 24.0 ± 4.8%) and small peptidergic neurons (identified by the co-labelling of GFP and substance P, 22.2 ± 1.7%) over non-peptidergic cells (identified by the co-labelling of GFP with IB_4_, 12.7 ± 1.7%). Interestingly, when mice were injected with the same adenovirus at 2 weeks of age, the infection rate of primary afferents was decreased by approximately half (9.2 ± 0.4% of total neurons, n = 5 mice). No GFP-positive cells were observed when mice were injected at the adult age (not shown; 6 mice injected 5 to 6 weeks after birth).

The infection induced by the intraplantar injection of AAV2/9-CBA-Cre-GFP appeared to be restricted to the DRGs, as GFP staining was virtually absent in the spinal cord of mice infected with AAV2/9-CBA-Cre-GFP alone in the hindpaws (Fig. 1C). However, tdTomato-positive fibers were abundant in the spinal cord of mice co-injected with emCBA-Flex-tdTomato-WPRE (2.6 × 10^13^ GC/ml) and AAV2/9-CBA-Cre-GFP (Fig. 1C), supporting the idea that the intraplantar injection of AAV2/9-CBA-Cre-GFP produces a selective infection of primary afferents. The latter observation also confirms that the Cre is transduced and functional in infected neurons. An absence of tdTomato labeling was noted when the emCBA-Flex-tdTomato-WPRE (2.6 × 10^13^ GC/ml) was injected alone (not shown), thus confirming the Cre dependency of the tdTomato expression.

**Figure 3:**
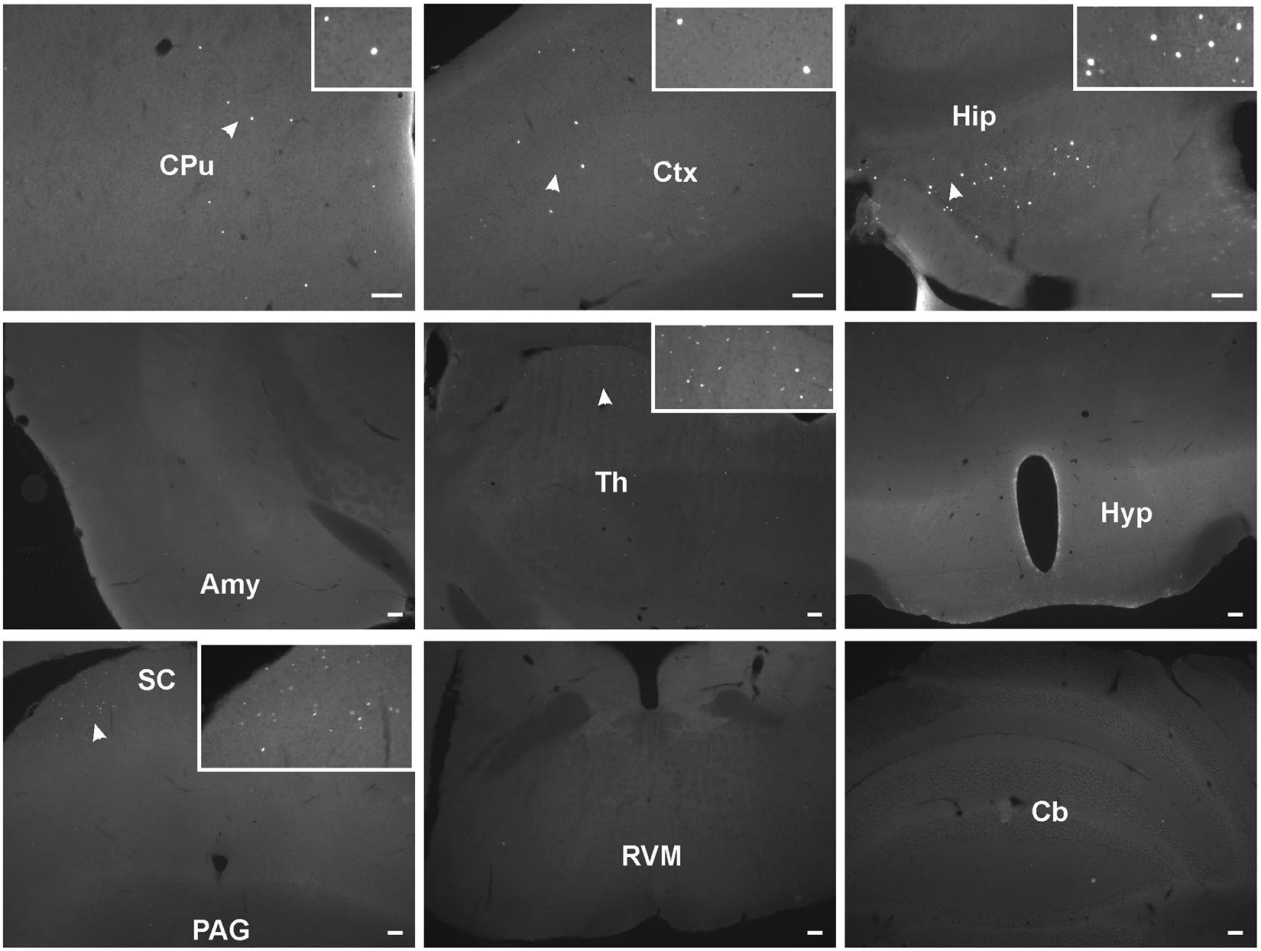
Distribution of GFP-positive cells in the central nervous system following intraplantar administration of AAV2/9-CBA-Cre-GFP at postnatal day 5. Representative photomicrographs illustrating GFP distribution in the brain of an animal injected with AAV2/9-CBA-Cre-GFP are shown. Images are representative of GFP staining observed in 3 mice. Amy: Amygdala, Cb: Cerebellum, CPu: Caudate putamen, Ctx: Cortex, Hip: Hippocampus, Hyp: Hypothalamus, PAG: Periaqueductal gray matter, RVM: Rostro-ventral medulla, SC: Superior Colliculus, Th: Thalamus. Scale bars = 100 µm.

A more thorough investigation revealed that a small but consistent number of GFP-positive cells were also found in a few regions of the brain following the intraplantar administration of AAV2/9-CBA-Cre-GFP at postnatal day 5 (Fig. 3). More specifically, a number of GFP-positive cells were observed in the hippocampus (Hip), cortex (Ctx), striatum (CPu) and superior colliculus (SC). Among the areas associated with pain processing, scattered GFP-positive cells were found lying in the dorsal part of the thalamus (Th). By contrast, no staining was detected in the rostroventral medulla (RVM), the locus coeruleus, parabrachial nuclei, the periaqueductal grey (PAG), nor in the amygdala (Amy) (Fig. 3).

### Intrathecal AAV infections

**Figure 4:**
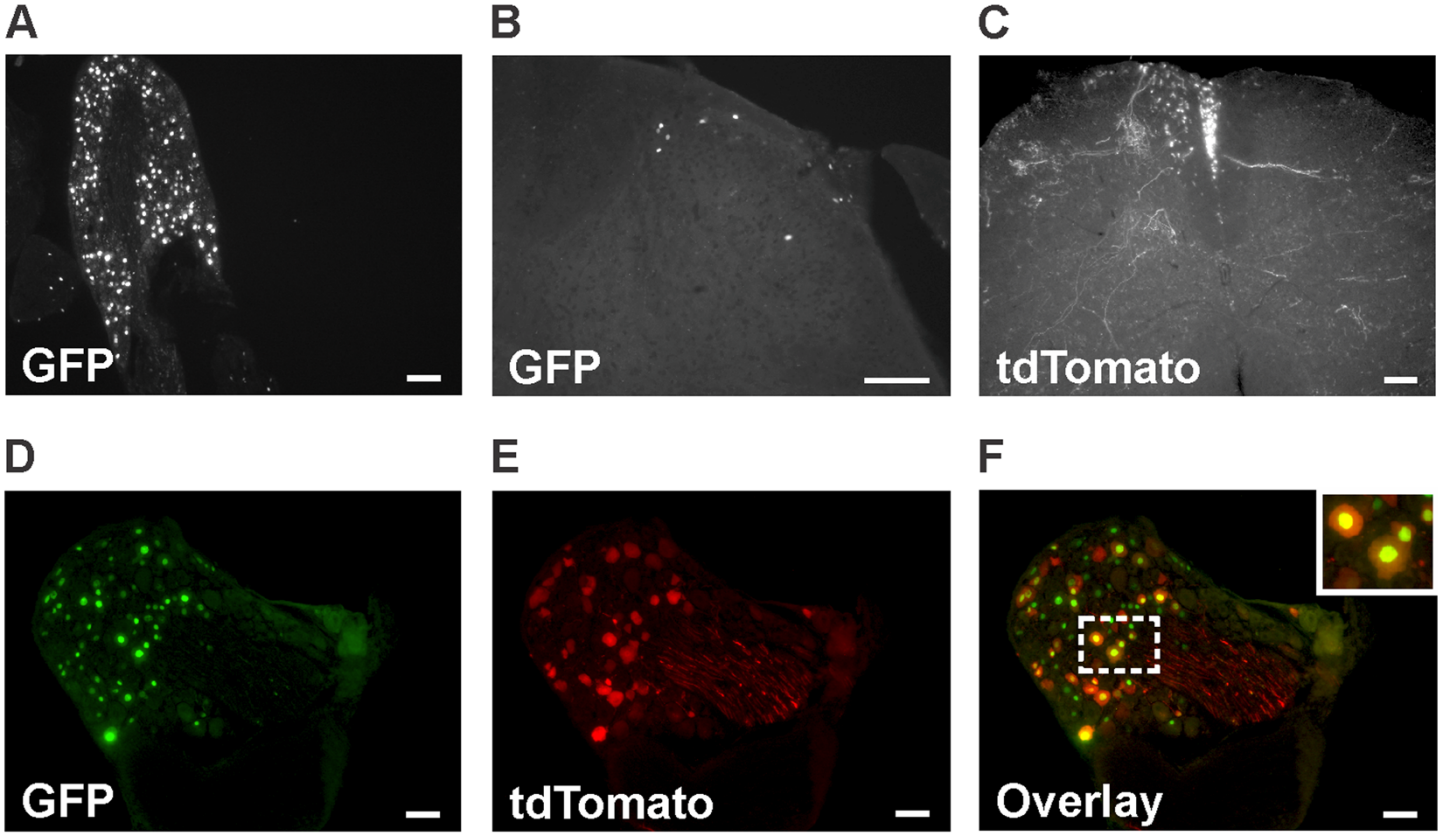
Distribution of GFP-positive cells in lumbar dorsal root ganglia and spinal cord following the intrathecal administration of AAV2/9-CBA-Cre-GFP. Representative photomicrographs illustrating GFP distribution in lumbar dorsal root ganglia (**A**) and the lumbar spinal cord (**B**, the dorsal horn is shown) of a mouse injected intrathecally with the AAV2/9-CBA-Cre-GFP virus at the adult age (25 days old; n = 4 mice). A representative photomicrograph illustrating the expression of tdTomato in the lumbar spinal cord of an animal co-injected with AAV2/9-CBA-Cre-GFP and AAV-emCBA-Flex-tdTomato-WPRE is also shown (**C**). (**D**–**F**) Nuclear GFP staining and tdTomato expression in a lumbar DRG of an animal co-injected with AAV2/9-CBA-Cre-GFP and AAV-emCBA-Flex-tdTomato-WPRE are shown. For panels C–F, images are representative of what has been observed in 3 mice. Scale bars = 100 µm.

Following the intrathecal administration of AAV2/9-CBA-Cre-GFP (1.6 × 10^13^ GC/ml) at 25 days of age, DRGs appeared as being highly infected (Fig. 4A). GFP staining was indeed present in 51.6% of L3-L5 DRG neurons (63.9 ± 8.5% in L3, 42.8 ± 7.4% in L4 and 48 ± 7.8% in L5; n = 4). A small number of GFP-positive neurons was also observed in the superficial laminae and in the white matter of the lumbar spinal cord (Fig. 4B). On average, 4.2 ± 1.6 GFP positive neurons were present in each lumbar spinal cord hemisection (these cells were located mostly in the superficial laminae). When mice were co-injected intrathecally with emCBA-Flex-tdTomato-WPRE AAV (2.6 × 10^13^ GC/ml), a high density of tdTomato-positive fibers was apparent in the spinal cord (Fig. 4C). In DRGs, cells expressing tdTomato were also found to be GFP-positive, further confirming the functionality of the Cre recombinase (Fig. 4D–F).

**Figure 5:**
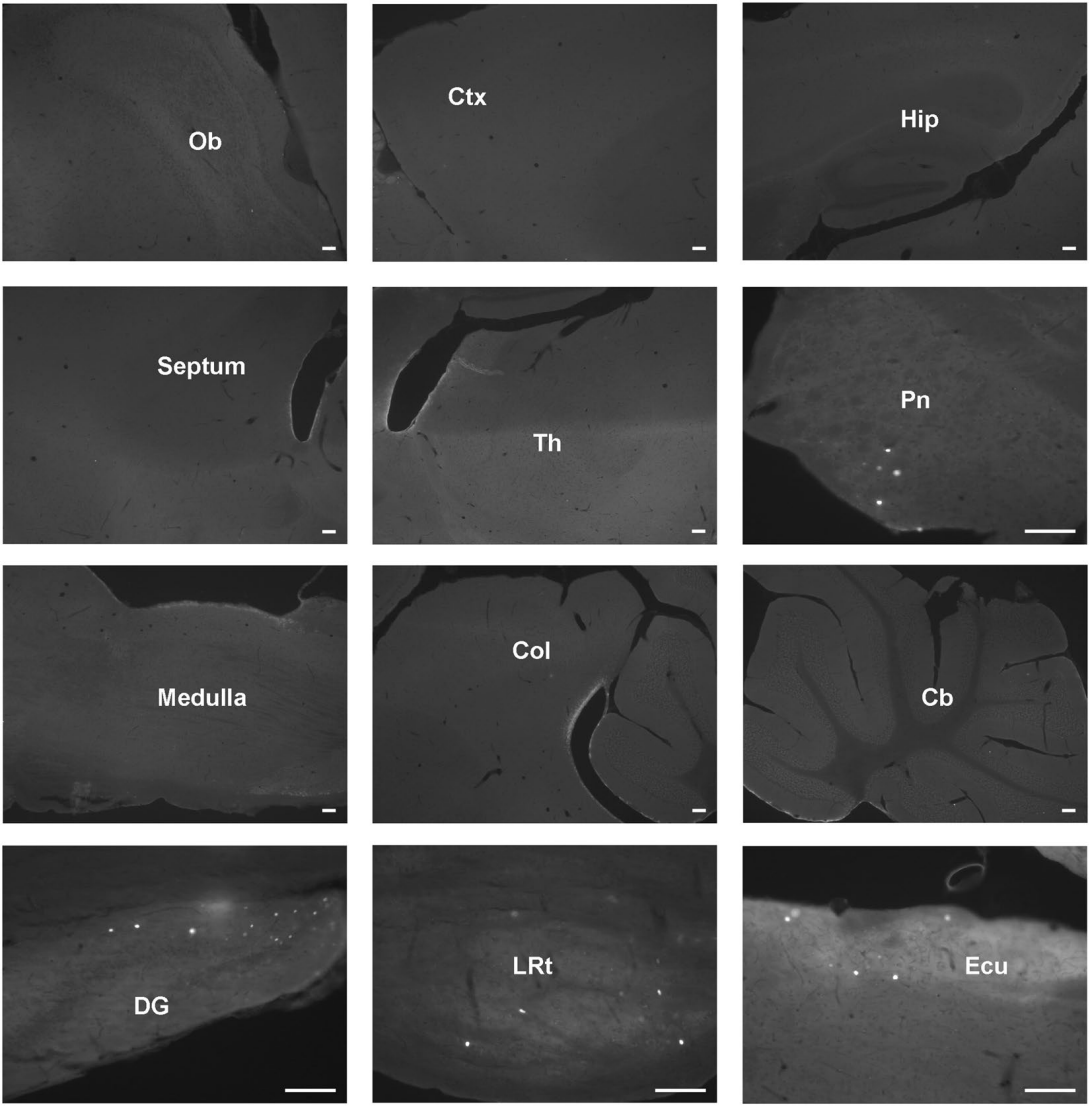
Distribution of GFP-positive cells in the brain following the intrathecal delivery of the AAV2/9-CBA-Cre-GFP virus. Representative photomicrographs of sagittal sections of the brain are shown (n = 3 mice). A few neurons expressing Cre-GFP 6 weeks after the intrathecal injection of the AAV2/9-CBA-Cre-GFP virus can be observed in various regions. Cb: Cerebellum, Col: Colliculus, Ctx: Cortex, DG: Dentate gyrus, ECu: External cuneate nucleus, Hip: Hippocampus, LRt: Lateral reticular nucleus, Pn: Pontine nucleus, Ob: Olfactory bulb, Th: Thalamus, Scale bars = 100 µm.

Compared to mice infected in the hindpaws, only discrete brain areas were found to have GFP-positive neurons following intrathecal administration of the AAV2/9-CBA-Cre-GFP adenovirus. Indeed, only scarce GFP-positive cells were observed in the pontine nucleus, lateral reticular nucleus and external cuneate nucleus (Fig. 5) while low number of GFP-positive cells were also apparent in the olfactory bulb, cortex, superior and inferior colliculus, as well as in the lateral lemniscus tract and in the cerebellum (not shown).

## Discussion

Recombinant adeno-associated viruses (rAAVs) are frequently used as a gene delivery strategy to either downregulate or rescue the expression of proteins and peptides *in vivo*^19–21^. This rAAV-mediated gene transfer represents a safe approach with minimal adverse effects to mediate long-term gene expression (for reviews see refs^4,5^).

In the present study, we aimed at specifically delivering the tyrosine recombinase enzyme Cre in mouse primary afferents. The Cre recombinase uses a topoisomerase I-like mechanism to catalyze the site-specific recombination of DNA between LoxP sites^2^. With the growing number of genetically modified rodent models and reporter mice using this system, the delivery of Cre to dorsal root ganglia (DRG) neurons represents a useful approach to turn “ON” or “OFF” the expression of various genes in these neurons, eliminating the need for multiple rounds of breeding with tissue-specific Cre-expressing mice (when available). In particular, this strategy could be useful, when combined to conditional knockin^22^ or conditional knockout mice^23–25^ to specifically re-express (under their endogenous promoter) or downregulate a peptide or a protein of interest in DRGs to further study their roles in pain and/or antinociception. To this end, the level of Cre expression is not an issue, as DNA recombination is permanent and only requires trace amounts of the enzyme. However, the level of infection, represented by the proportion of infected neurons, needs to be maximal.

Direct injections of rAAVs into the DRG or in the sciatic nerve was previously described as a suitable approach for the selective gene-transfer into primary sensory neurons^11,15,18^. However, these approaches require specialized technical skills and are invasive, as they require surgery to expose the nerve or the ganglion. Here, we used intraplantar and intrathecal injections as potential alternative routes of delivery and compared their ability and efficacy to selectively transfer a functional GFP-tagged version of Cre in primary afferents. The rAAV2/9-CBA-Cre-GFP virus was selected to allow the visualization and characterization of the infected cells in the nervous system. It is also worth noting that transduction efficacy in primary afferents was shown to be highly dependent on the virus’ serotype^14,15,26^. For instance, it was observed that AAV8 has a much higher transduction rate in primary afferents than does AAV1^14^. As for AAV9, it was previously used to efficiently transduce primary afferents via intrathecal delivery^8,12,17,27^.

When injected intrathecally, we found that the rAAV2/9-CBA-Cre-GFP virus exhibited a significantly higher rate of gene-transfer in lumbar DRGs when compared to intraplantar delivery. The high rate of transduction observed following the intrathecal delivery of the rAAV2/9-CBA-Cre-GFP virus is comparable to previous observations via this route of administration^11–13^. However, the reason why intraplantar- is less efficient at infecting DRG neurons than intrathecal-mediated infection remains unclear. Peripheral free nerve endings may be less prone or more resistant to virus entry than central synapses, which may explain the lower infection rate obtained by this route of infection. In short, it is possible that nerve endings, in the periphery, do not easily uptake viruses because they are less accessible or simply more resistant to infections than central terminals. As illustrated by the relatively high rate of gene-transfer in other DRGs and in trigeminal ganglia neurons, the peripherally-injected virus likely exits the site of injection and has therefore the capability of infecting all primary afferents rather than only those located around the site of injection. Furthermore, the age-dependency of peripheral viral infection efficacy can also be an issue. Similar to previous findings^7–9^, it was also found that the number of GFP-positive DRG neurons was greatly reduced when the intraplantar infection was performed at postnatal day 15 (as compared to postnatal day 5). As shown by others^8^, we cannot rule out the possibility that a higher rate of infection would have been observed if we had performed the intraplantar infection in neonates. The use of neonatal mice however presents other limitations such as size of the animal and the immaturity of the primary afferents. For example, it is known that TRPV1 has a widespread expression in DRGs of neonates and that the specialization of these neurons follows developmental stages^28^. Therefore, the expression of Cre driven by a specific promoter (e.g. TRPV1 promoter) at early developmental stages may introduce recombination in neurons where that specific promoter is inactive at the adult age.

The AAV9 serotype was previously found to cross the blood-brain barrier when injected intravenously (i.v.) leading to the infection of neurons throughout the central nervous system^12,29^. Consequently, such a wide distribution of the virus/infection would reduce the usefulness of this tool to specifically target DRG neurons. In the current study, only few brain regions were found to express GFP after either intraplantar or intrathecal administration of the rAAV2/9-CBA-Cre-GFP virus. With regards to pain research, it was interesting to find that pain-related structures other than the thalamus, when the virus was administered in the hindpaws, were free from any GFP labeling. Thus, since we relied on the GFP signal to identify infected cells, one could argue that the level of GFP in the brain or in the spinal cord was too low to be directly detected. Another possible factor that might have affected the level of Cre-GFP expression could be that the animals were euthanized between 8 to 12 weeks after the viral infection. When compared to the neuron specific enolase (NSE) or to the cytomegalovirus early enhancer (CMV) promoters, the hybrid cytomegalovirus enhancer/chicken β-actin (CBA) promoter used here produces a robust and long-term gene expression^30,31^. Additionally, we have used anti-GFP antibodies to amplify further the fluorescent labeling in cells expressing lower levels of Cre-GFP. We are therefore confident that the percentage of infected GFP-positive neurons identified here supports the idea that both routes of administration of the rAAV2/9-CBA-Cre-GFP virus predominantly transduce primary afferents. When injected intrathecally, the rAAV2/9-CBA-Cre-GFP virus only infected a few spinal neurons. The fact that only tdTomato-positive projections and not cell bodies were observed in the spinal cord of mice co-infected with emCBA-Flex-tdTomato-WPRE and rAAV2/9-CBA-Cre-GFP viruses also supports this conclusion. In agreement with our observations, an absence or a very limited GFP distribution across the brain was previously reported following the intrathecal delivery of AAV9 and AAV8 viruses^14,32^. This finding however sharply contrasts other observations which reported a wide distribution of GFP in the brain 6 weeks following infection with either AAV9 or AAV5^12,33^.

Overall, this study reveals that the intrathecal administration of the rAAV2/9-CBA-Cre-GFP virus represents an efficient and non-invasive strategy to selectively deliver a Cre recombinase to DRG neurons. Since intrathecal injections can be easily performed in adult mice with minimal loss of infection efficacy, problems related to developmental differences in gene expression can be avoided. Given the high efficiency of infection and the fact that trace amounts of Cre recombinase are necessary to catalyze the permanent recombination of DNA, one might also use specific promoters (e.g. TRPV1 promoter) to selectively target a subpopulation of DRG neurons in order to drive the expression of Cre.

## Material and Methods

### Animals

Newborn and adult mice used in this study were produced in our own colony (which is backcrossed twice a year with C57BL/6 mice from Charles River, St-Constant, QC, Canada). All animals were maintained in a light and temperature controlled environment (Light on at 6AM–8PM, room temperature 22 °C) with constant access to water and food. All procedures were approved by the animal care committee of the Université de Sherbrooke (protocol # 242-14) and were in accordance with the ethical guidelines of the Canadian council on animal care (CCAC) and the International Association for the Study of Pain (IASP).

### AAV infection

Two groups of mice were used. In the first group, conscious, non-anesthetized mice received intraplantar injections (5 µl in each hindpaws) at either postnatal day 5, 2 weeks of age, or at an adult age (5–6 weeks). In the second group, conscious, non-anesthetized mice received intrathecal injections (5 µl) of a recombinant adeno-associated virus combining a serotype 2 replicase and serotype 9 capsid (AAV2/9) engineered to express a chimeric fluorescent Cre recombinase (Cre-GFP) under the chicken beta actin promoter (CBA) (AAV2/9-CBA-Cre-GFP; 1.25–1.6 × 10^13^ GC/ml, purchased from the Molecular Tools Platform, Université Laval, Québec, QC, Canada) 25 days after birth. For the co-injection experiments, 2.5 µl each of AAV2/9-CBA-Cre-GFP (1.25–1.6 × 10^13^ GC/ml) and AAV-emCBA-Flex-tdTomato-WPRE (an AAV engineered to express, under the enhanced mini Chicken beta-actin promoter, the fluorescent tdTomato flanked with LoxP recombination sites; 2.6 × 10^13^ GC/ml, Molecular Tools Platform, Université Laval) were mixed and injected in both hindpaws of postnatal day 5 mice or intrathecally to 25 days old mice. Two to three months later, animals were euthanized and tissues collected for immunohistochemical processing (see below).

### Immunofluorescence

Animals were anesthetized with a mixture of isoflurane and medical air and perfused with 5 mL of 0.9% heparinized saline solution followed by 30 mL of 4% PFA in a phosphate buffer solution (0.1 M PBS). Fixed tissues (spinal cord, brain, ganglia) were then removed and post-fixed in 4% PFA/PBS for 1–2 h before being cryoprotected with a 30% sucrose solution. The whole brain, lumbar spinal cord, trigeminal ganglia (TGs) and dorsal root ganglia (DRGs) were then sectioned at a thickness of 20 (ganglia) or 30 (brain and spinal cord) microns using either a cryostat (Leica CM1860, Leica Biosystems Inc., Concord, ON, Canada) or a freezing microtome (Leica SM2000R, Leica Biosystems Inc.).

Sections from lumbar DRGs mounted on gelatin-coated slides were incubated overnight at 4 °C with a 1/500 dilution of rabbit anti-GFP antibody (Millipore cat. AB3080, Etobicoke, ON, Canada or GeneTex cat. GTX30266, Atlanta, GA, USA), 1/1000 of biotin-conjugated isolectin B_4_ (IB_4_; Sigma Aldrich, cat. L-2140, Oakville, ON, Canada), 1/750 guinea pig anti-Substance P (Neuromics, cat. GP14103, Edina, MN, USA) or 1/1000 mouse anti-NF200 (Sigma Aldrich, cat. N0142) in 0.1 M PBS containing 0.3% Triton X100, 3% NGS and 1% BSA. Primary antibodies were detected using 1/500 Alexa 488 goat anti-rabbit, 1/1000 streptavidin Alexa 546, 1/500 Alexa 405 goat anti-mouse or 1/500 Alexa goat anti-guinea pig (secondary antibodies were purchased from Life Technologies, Thermo Fisher Scientific, Burlington, ON, Canada). After sections were washed with PBS and air-dried, coverslips were mounted using Aqua Polymount medium (Polysciences Inc., cat. 18606, Warrington, PA, USA). All images were acquired using a Leica DM4000B epifluorescence microscope and a Leica DFC350FX monochrome camera or with a Hamamatsu NanoZoomer 2.0-RS slide scanner (Leica Biosystems).

### Counting

Counting was done using the ImageJ open source Software (National Institute of Health, Bethesda, MD, USA) on images of successive sections of lumbar DRGs (8–18 sections per mice) or spinal cords (3–5 sections per mice). Results are expressed as the mean number of cells per section averaged for “n” animals. Note that because Cre is a nuclear enzyme, the GFP signal was only observed in the nucleus of cells.
